# Supplementary material for: Can the current radiation dose, in chest tomosynthesis, be reduced with retained image quality? A study in the context of lung cancer screening programs
Source: PLoS One. 2026 Mar 16;21(3):e0343760. doi: 10.1371/journal.pone.0343760 (PMC12991223; doi:10.1371/journal.pone.0343760)
Supplement: Attachment — Submitted filename: Response to Reviewers.docx [file pone.0343760.s002.docx]

# Journal Requirements:

***2. We note that you have indicated that there are restrictions to data sharing for this study. For studies involving human research participant data or other sensitive data, we encourage authors to share de-identified or anonymized data. However, when data cannot be publicly shared for ethical reasons, we allow authors to make their data sets available upon request. For information on unacceptable data access restrictions, please see*** [***http://journals.plos.org/plosone/s/data-availability#loc-unacceptable-data-access-restrictions***](http://journals.plos.org/plosone/s/data-availability#loc-unacceptable-data-access-restrictions)***.*

*Before we proceed with your manuscript, please address the following prompts:

a) If there are ethical or legal restrictions on sharing a de-identified data set, please explain them in detail (e.g., data contain potentially identifying or sensitive patient information, data are owned by a third-party organization, etc.) and who has imposed them (e.g., a Research Ethics Committee or Institutional Review Board, etc.). Please also provide contact information for a data access committee, ethics committee, or other institutional body to which data requests may be sent.***

**Author:** The data used in this study contain potentially identifying and sensitive patient information, including patient names, patient identification numbers, and other confidential clinical details. For this reason, full access to the dataset is restricted. Approval for the study was obtained from the Regional Ethical Review Board in Stockholm, Sweden. The data are owned and controlled by Karolinska University Hospital, Solna, in collaboration with Karolinska Institutet, Stockholm. These institutions impose the restrictions on data sharing to ensure the protection of patient privacy and compliance with Swedish and EU ethical and data-protection regulations.

Data may be made available to qualified researchers who meet the criteria for access to confidential data upon reasonable request to the institutional contact person, Dr. Sven Nyré, and subject to approval by the relevant institutional bodies.

Dr. Sven Nyré
Karolinska University Hospital, Solna / Karolinska Institutet
Department of Radiology, Karolinska University Hospital, Solna- Sweden
Email: sven.nyren@regionstockholm.se
Phone: +46(0)702984725

Please note that any data sharing will require appropriate ethical approval and a data-sharing agreement with the data-owning institutions.

***b) If there are no restrictions, please upload the minimal anonymized data set necessary to replicate your study findings to a stable, public repository and provide us with the relevant URLs, DOIs, or accession numbers. Please see*** [***http://www.bmj.com/content/340/bmj.c181.long***](http://www.bmj.com/content/340/bmj.c181.long) ***for guidelines on how to de-identify and prepare clinical data for publication. For a list of recommended repositories, please see*** [***https://journals.plos.org/plosone/s/recommended-repositories***](https://journals.plos.org/plosone/s/recommended-repositories)***. You also have the option of uploading the data as Supporting Information files, but we would recommend depositing data directly to a data repository if possible.

Please update your Data Availability statement in the submission form accordingly.***

**Author:** The dataset containing imaging protocol settings, de-identified patient data, corresponding dose calculations, ViewDEX configuration files, and the observers’ evaluation results have been made publicly available in the Figshare repository (<https://figshare.com/>), where it has been assigned the DOI: **httpsdoi.org10.6084m9.figshare.30621287**

***3. Please ensure that you refer to Figure 1 in your text as, if accepted, production will need this reference to link the reader to the figure.***

**Author:** As requested, the ‘Examination workflow section has been updated to include an explicit reference to Figure 1

***Additional Editor Comments:
The paper talks about an interesting subject in radiology and imaging. The discussion however needs to be strengthened by synthesizing how findings from this study can be useful to a more global audience beyond the setting where the study was conducted from. In addition, the entire paper needs proof-reading to improve on the language.***

**Author:** Please see the respond to the editor under section “Discussion”.

# Reviewer #1

1. ***Acquisition Physics and Dose-Reduction Mechanism:
   The reduced-dose protocols were achieved by adding 20 mm aluminium filtration and lowering the tube voltage to 100 kV, while the vendor-recommended protocol is reported as 125 kV. Please clarify this inconsistency (100 kV vs 120 kV/125 kV) and quantify how the added filtration affected beam quality (e.g., half-value layer, mean photon energy). Including measured or simulated x-ray spectra and HVL data would help validate that image-quality changes are not confounded by spectral shifts rather than dose effects.***

**Author:** The vendor-recommended acquisition protocol (without additional aluminum filtration) uses a tube voltage of 120 kV. In this study, all three tomosynthesis protocols were instead acquired at 100 kV, combined with an added 20 mm aluminum filter, and this 100 kV + 20 mm Al setting was applied consistently for tomosynthesis acquisitions.

An additional 20 mm aluminum filtration was implemented identically for all three tomosynthesis protocols, resulting in comparable radiation quality across these settings. The resulting beam quality, including the mean photon energy, was therefore effectively the same for all tomosynthesis acquisitions, so the differences observed between protocols are not attributable to changes in spectral quality but to differences in dose and acquisition geometry.

A spectrum processor was used to simulate the X‑ray spectra (Siemens Healthiness, Spectrum Simulation Tool). The mean photon energy of the original spectrum (120 kV, 13 mm Al, 0.1 mm Cu) was 67.4 keV and for the new protocol (100 kV, 33 mm Al, 0.1 mm Cu), the mean photon energy was 67.5 keV.

2. ***Dose Estimation and Method Transparency
   You report that effective dose was calculated from the dose-area product (DAP) of the scout image, scaled by projection ratios and a field-size factor (0.935).
   Please detail how these scaling factors were derived, cite supporting references, and provide per-protocol DAP and effective-dose statistics (mean ± SD or median [IQR]) in a summary table. Consider stratifying by patient size or BMI to demonstrate robustness across different body habitus.***

**Author:** Thank you for pointing out the lack of clarity in this section. In this response, I describe the method in more detail, while in the revised manuscript I have included a shorter, summarized version to improve readability.

For the VolumeRAD chest tomosynthesis system, Båth et al. determined an average effective dose including scout and 60 projections was about 0.13 mSv for a standard‑sized adult (≈170 cm, 70 kg), together with an average total DAP of 0.47 Gy·cm² for the tomosynthesis acquisition. To facilitate estimation of patient radiation dose in chest tomosynthesis, a conversion coefficient of (0.26 mSv/(Gy·cm²) introduced that links the total DAP of the tomosynthesis examination to the corresponding effective dose. This conversion coefficient was obtained for a standard‑sized adult patient using Monte Carlo simulations of the VolumeRAD system (GE Healthcare, Chalfont St. Giles, UK), which at the time was the most extensively evaluated platform for both scientific and clinical studies of chest tomosynthesis. It showed that the same coefficient (0.26 mSv/(Gy·cm²)) applies to the PA projection and the scout view in this system, indicating that dose estimates for chest tomosynthesis with VolumeRAD can be based directly on the total measured DAP without analyzing each individual projection. ^Båth_Radiation-Protection-Dosimetry 2010; Båth_Med Phys. 2014^

A method described by Båth et al. enables estimation of the DAP for a VolumeRAD chest tomosynthesis examination directly from chest tomosynthesis data contained in the scout image, and this approach has been developed and validated for clinical use. In the method chest tomosynthesis data from 20 patients were used to link the scout image to the full tomosynthesis acquisition. For each examination, the AEC‑determined tube load of the scout was scaled by the system dose ratio of 10, divided by 60, rounded down to the nearest step with a lower limit of 0.25 mAs, and then multiplied by 60 to give an estimated total tube load for the tomosynthesis series. The initial estimate used the scout to predict the total DAP while temporarily ignoring how the field size changes with projection angle. To correct for this, Båth et al. compared the scout‑based DAP estimate with the true total DAP from all 60 projections for each patient and fitted a straight line through the origin. The slope of this line was then used as the ^Båth_Med Phys. 2014^

Table 1: Summary of dose-area product (DAP), effective dose, and percentage of effective dose in relation to ratio 10, estimated across body mass index (BMI) groups using different ratios.

|  |  |  | **Ratio 10** | | **Ratio 7** | | | **Ratio 5** | | |
| --- | --- | --- | --- | --- | --- | --- | --- | --- | --- | --- |
| BMI | Average DAP value  dGy/cm2 | SD- DAP value | Average effective Dose **(Ratio 10)** mSv | SD effective dose **(Ratio 10)** | Average Effective Dose **(Ratio 7)** mSv | Percentage of effective dose in relation to ratio 10 | SD effective dose **(Ratio 7)** | Average Effective Dose **(Ratio 5)** mSv | Percentage of effective dose in relation to ratio 10 | SD effective dose **(Ratio 5)** |
| <25 | 2,53 | 0,73 | 0,56 | 0.03 | 0,39 | 70% | 0.13 | 0,29 | 52% | 0.10 |
| 25-30 | 4,11 | 1,03 | 0,9 | 0.04 | 0,6 | 67% | 0.14 | 0,43 | 48% | 0.11 |
| >30 | 4,78 | 0,57 | 1,01 | 0.03 | 0,63 | 62% | 0.22 | 0,45 | 45% | 0.18 |

In this cohort, 52% of participants had BMI <25, 36% BMI 25–30, and 12% BMI >30, indicating a broad distribution of body habitus. Table 1 shows that both DAP and effective dose increase with BMI, with mean DAP values of 2.53, 4.11, and 4.78 dGy/cm² and corresponding effective doses of 0.56, 0.90, and 1.01 mSv when using ratio 10. Effective doses are lower when using ratios 7 and 5 but follow the same BMI‑dependent pattern, confirming higher BMI is generally associated with greater radiation exposure, consistent with the need for increased dose in patients with larger body thickness. This trend is confirmed by the percentage of effective dose in relation to ratio 10, with slightly different values across the BMI groups. The effective dose conversion 0.26 mSv/Gycm^2^ was used for all ration factors and all BMI groups.

***3. Statistical Reporting: Precision, Readers, and Multiplicity:
Provide 95% confidence intervals for AUC_{VGC} values and include effect sizes alongside p-values. Also, Quantify inter-observer agreement (e.g., ICC) to assess consistency between readers. You may consider a multi-reader, multi-case (MRMC) analysis or mixed-effects model to appropriately handle correlated data. Finally, discuss whether corrections for multiple testing across the four quality domains were applied.***

**Author:** In VGC analysis, which is a multi-reader, multi-case (MRMC) analysis, statistical uncertainty is handled by bootstrapping the pooled ordinal scores over all observers and cases. Correlated data are thus appropriately handled, similarly to MRMC ROC analysis. The resulting VGC curve represents the average ratings, and the asymmetric 95% confidence interval for AUC_VGC_ is estimated via resampling. The p-value is calculated as the fraction of bootstrap samples producing results equal to or more extreme than the null hypothesis (AUC_VGC_ = 0.5).^Hansson_RPD 2021^

The effect size for each comparison was quantified by calculating the absolute difference in AUC between each dose-reduced protocol and the standard dose protocol which is 0.5. This effect size demonstrates not only whether differences were statistically significant but also their magnitude, clarifying that observed changes in image quality are statistically detectable but relatively small overall.

Table 2. The result of the comparison between vendor recommended protocol and the protocol with 30% and 50% dose reduction respectively by using VGC Analyzer

| DTS low dose acquisitions | **Vessel demarcation** | | | **Vessel disturbance** | | | **Bone structure** | | | **Tumour homogeneity** | | |
| --- | --- | --- | --- | --- | --- | --- | --- | --- | --- | --- | --- | --- |
|  | AUC_VGC_ | *p*-value | Effect size | AUC_VGC_ | *p*-value | Effect size | AUC_VGC_ | *p*-value | Effect size | AUC_VGC_ | *p*-value | effect size |
| Tomosynthesis acquisition 2 with 30% dose reduction | 0.51 | 0.57 | 0.01 | 0.51 | 0.59 | 0.01 | 0.50 | 0.88 | 0 | 0.47 | 0.09 | 0.03 |
| Tomosynthesis acquisition 3 with 50% dose reduction | 0.47 | 0.03 | 0.03 | 0.47 | 0.02 | 0.03 | 0.50 | 0.79 | 0 | 0.46 | 0.10 | 0.04 |

Just as in MRMC ROC analysis, no comparison between the direct ratings made by the observers is made in VGC analysis. Instead, the ratings from a given observer are used to determine an AUC for this observer, and the final AUC is obtained by averaging the AUCs across observers. There are several reasons for this approach, one being that it is not possible to know whether a difference in ratings between observers is due to differences in interpretation of the rating scale or differences in assessment of the image quality. Thus, determining inter-observer agreement based on the given ratings is not meaningful. For example, if observer A gives a rating of 1 to all cases from modality A and a rating of 2 to all cases from modality B, whereas observer B gives a rating of 2 to all cases from modality A and a rating of 3 to all cases from modality B, an agreement analysis based on for example the kappa value would indicate that there is no agreement between the observers. However, the observers are in fact in perfect agreement in that they both always rate modality B higher.

VGC analysis, as implemented in both the VGC Analyzer software and the bootstrapping methods described in the literature, is specifically designed for multi-reader, multi-case (MRMC) studies. In VGC analysis, multiple observers (readers) evaluate multiple images (cases) for different protocols, and the statistical inference (AUCVGC, confidence intervals, p-values) is based on the combined ordinal data across all readers and cases. The bootstrapping and resampling approaches used in VGC Analyzer explicitly account for reader and case variability, enabling robust analysis in fully crossed MRMC experimental designs. Published comparisons have shown that VGC analysis produces results closely comparable to established gold-standard ROC MRMC methodologies when analyzing paired and unpaired multi-reader data. ^Hansson_RPD 2021^

Therefore, the methodology used in this study already addresses correlated data through the MRMC structure of VGC analysis and its associated resampling-based uncertainty estimation. Additional mixed-effects modelling would not provide new information beyond the established VGC approach for these ordinal quality ratings.

4. ***Link to Clinical Performance and Population:
   As your cohort consists of patients with known or suspected malignancy rather than a screening population, please temper the generalization to screening settings. If feasible, include an exploratory analysis of lesion visibility stratified by size (e.g., >10 mm vs <10 mm) using CT as the reference. At minimum, discuss how trends in tumor-homogeneity scores may translate to detection or characterization performance.***

**Author:**

- The text in the section “Discussion” has been updated accordingly: DTS technique could be adopted to being included in a lung cancer screening program as complementary imaging technique.
- Assessment of lesion size will be performed in a forthcoming study, whose data are presently under review

Table 2 shows that tumor-homogeneity scores (AUC_VGC_ values) remain close to 0.50 across all participants (all BMI groups) and acquisition protocols, and no statistically significant differences were found between the standard dose protocol and protocols with reduced dose. This means that within this study population, variations in radiation dose and BMI did not materially affect the perceived uniformity or structural assessment of tumors on digital tomosynthesis images.

Stable tumor-homogeneity scores indicate that pour image quality due to dose reduction is minimal for the specific task of visualizing tumor structure and texture. As homogeneity is crucial for recognizing abnormal tissue patterns in the background of the lung, consistent scores across protocols imply that diagnostic performance for basic tumor detection is likely maintained, even at lower doses. However, subtle differences in very low contrast or heterogeneous neoplasms may still require confirmation by higher-resolution modalities such as CT.

5. ***Observer Study Details and Reproducibility:
   Clarify the number of rated items per class, treatment of any missing ratings, randomization order, and the contents of the training set.
   Indicate whether reading order was balanced across protocols. And, providing your ViewDEX configuration files and full rating criteria as supplementary materials would enhance reproducibility.***

**Author:** As requested, the ‘Observers’ assessment of image quality’ section has been updated to address these points.

The image quality ratings were based on a total of 14 questions, which included 5 about vessel demarcation, 5 concerning vessel disturbance, 2 related to bone structure, and 2 focused on tumor homogeneity (Table 1). No ratings were missing. All observers completed the full set of questions for each case. In ViewDEX, the image series are displayed in a unique random order for each observer along with a set of criteria, and the responses of the observer (the given rating for each criterion) are registered and stored in a log file for analysis. The randomization of image presentation order is managed automatically by ViewDex software, ensuring the sequence assignment is free from human bias. Prior to the main evaluation, all observers received standardized instructions and reviewed a training set of representative test cases, which ensured familiarity with the rating criteria and evaluation procedure. This approach aligns with best practices in observer performance studies to ensure unbiased and reproducible results.

ViewDEX configuration files, and rating criteria have been made publicly available in the Figshare repository (<https://figshare.com/>), where it has been assigned the DOI: **httpsdoi.org10.6084m9.figshare.30621287**
