# Supplementary material for: Can the current radiation dose, in chest tomosynthesis, be reduced with retained image quality? A study in the context of lung cancer screening programs
Source: PLoS One. 2026 Mar 16;21(3):e0343760. doi: 10.1371/journal.pone.0343760 (PMC12991223; doi:10.1371/journal.pone.0343760)
Supplement: Attachment — Submitted filename: Response_to_Reviewers_auresp_2.docx [file pone.0343760.s003.docx]

**Response to Reviewers**

**ACADEMIC EDITOR: 1. The discussion should show some practical implication of your findings in a broad sense such that even readers beyond your institution can get some benefit from the study.**

**Author:** The text in the section “Discussion” has been updated accordingly:

Our study supports that a dose reduction of 30%, resulting in a dose to the patient of 0.09 mSv per patient, does not lower image quality. A further reduction to 50%, with a dose to the patient of 0.06 mSv, slightly affects image quality, which might be acceptable in a screening context. The dose in CT screening is usually 1–1.5 mSv, thus 10–15 times higher than a screening with tomosynthesis would be. As a large number of healthy individuals are irradiated for every case of cancer that is found, the dose is of importance in screening programs.

Unlike previous DTS dose-reduction and nodule-detection studies that focused primarily on discrete nodules or limited regions, the current study is the first to prospectively compare three DTS protocols (30% and 50% dose reduction vs the vendor-recommended protocol) in patients with pulmonary malignancies, using multi-reader VGC analysis across thoracic regions. In contrast to earlier studies focused mainly on nodule detectability^4,7,33,37,68,69^, our observers graded lung parenchyma and vessels over a wide anatomical extent, including basal, retrocardiac, ventral and peripheral regions, as well as bone structures and tumor homogeneity. This design captures dose effects on overall thoracic image quality rather than on isolated nodules alone, which is crucial if DTS is to be used for interval follow-up and in lung cancer screening programs.

In addition, previous DTS studies, including that of Söderman^40^, have mainly assessed dose reduction using simulated noise or phantoms^41,70-72^, whereas the current patient study focuses on the systematic visual grading of thoracic image quality at different dose levels. Furthermore, this study systematically investigated pulmonary nodules of all types, not only round nodules, as in many earlier studies, but also irregular lesions with a wide range of shapes and considerable variation in size.

Asplund et al.^41^ showed that pulmonary nodule detectability remained unchanged when the effective dose was reduced to approximately 32% of the default setting using noise‑simulated tomosynthesis images, which aligns with our finding that diagnostic performance can be maintained despite substantial dose reduction in thoracic DTS. Meltzer et al.^63^ later demonstrated that DTS can be used for surveillance of small pulmonary nodules, although measurement variability limits precise growth assessment. In contrast, our prospective patient study directly compares three DTS dose protocols (30% and 50% dose reduction vs the vendor-recommended protocol) and shows that a 30% dose reduction does not degrade observer‑rated image quality for vessel demarcation, vessel disturbance, bone structures or tumor homogeneity, while a 50% reduction only slightly worsens vessel‑related criteria. These findings extend the existing literature by indicating that routine DTS dose can be lowered towards conventional chest radiography levels without sacrificing clinically relevant image quality, which is particularly important if DTS is to be implemented as a low‑dose, low‑cost follow‑up modality in lung cancer screening pathways and in settings where CT capacity and resources are limited.

Replacing LDCT examinations with low-dose DTS in lung cancer screening programs would reduce the radiation dose received by healthy participants by at least 90%. It would also considerably lower costs, as DTS examinations are much faster to perform, and the X-ray equipment is significantly less expensive, an important factor for countries with limited resources or remote areas.

**ACADEMIC EDITOR: 2. How do the findings from your study contribute to known literature around this area?**

**Author:** The text in the section “Discussion” has been updated accordingly.

Unlike previous DTS dose-reduction and nodule-detection studies that focused primarily on discrete nodules or limited regions, the current study is the first to prospectively compare three DTS protocols (30% and 50% dose reduction vs the vendor-recommended protocol) in patients with pulmonary malignancies, using multi-reader VGC analysis across thoracic regions. In contrast to earlier studies focused mainly on nodule detectability^4,7,33,37,68,69^, our observers graded lung parenchyma and vessels over a wide anatomical extent, including basal, retrocardiac, ventral and peripheral regions, as well as bone structures and tumor homogeneity. This design captures dose effects on overall thoracic image quality rather than on isolated nodules alone, which is crucial if DTS is to be used for interval follow-up and in lung cancer screening programs.

In addition, previous DTS studies, including that of Söderman^40^, have mainly assessed dose reduction using simulated noise or phantoms^41,70-72^, whereas the current patient study focuses on the systematic visual grading of thoracic image quality at different dose levels. Furthermore, this study systematically investigated pulmonary nodules of all types, not only round nodules, as in many earlier studies, but also irregular lesions with a wide range of shapes and considerable variation in size.

Asplund et al.^41^ showed that pulmonary nodule detectability remained unchanged when the effective dose was reduced to approximately 32% of the default setting using noise‑simulated tomosynthesis images, which aligns with our finding that diagnostic performance can be maintained despite substantial dose reduction in thoracic DTS. Meltzer et al.^63^ later demonstrated that DTS can be used for surveillance of small pulmonary nodules, although measurement variability limits precise growth assessment. In contrast, our prospective patient study directly compares three DTS dose protocols (30% and 50% dose reduction vs the vendor-recommended protocol) and shows that a 30% dose reduction does not degrade observer‑rated image quality for vessel demarcation, vessel disturbance, bone structures or tumor homogeneity, while a 50% reduction only slightly worsens vessel‑related criteria. These findings extend the existing literature by indicating that routine DTS dose can be lowered towards conventional chest radiography levels without sacrificing clinically relevant image quality, which is particularly important if DTS is to be implemented as a low‑dose, low‑cost follow‑up modality in lung cancer screening pathways and in settings where CT capacity and resources are limited.

**ACADEMIC EDITOR: 3. The paper needs proof-reading to improve the language**

**Author:** We appreciate the editor’s feedback regarding the language of the manuscript. We have carefully reviewed and revised the text to improve clarity and readability. In addition, we have asked a native English-speaking consultant with long experience in chest tomosynthesis research and in reviewing scientific articles in this field, and he has assisted with a thorough language review of the manuscript. If PLOS ONE could specify in more detail which aspects of the language require further attention, we would be pleased to address these points specifically, with his assistance.
